# Study on the Corrosion Mechanism of N80 Steel in Simulated Oxygen-Reduced Air Drive Production Wellbores

**DOI:** 10.1021/acsomega.3c02869

**Published:** 2023-06-25

**Authors:** Baocheng Shi, Xingwen Wang, Kaili Zhou, Kun Xue, Kai Liu, Xingkai Zhang, Yijie Qiu

**Affiliations:** † Hubei Key Laboratory of Drilling and Production Engineering for Oil and Gas, Yangtze University, Wuhan 430100, China; ‡ Oil and Gas Storage and Transportation Engineering Research Center (Hubei Province), Wuhan 430100, China

## Abstract

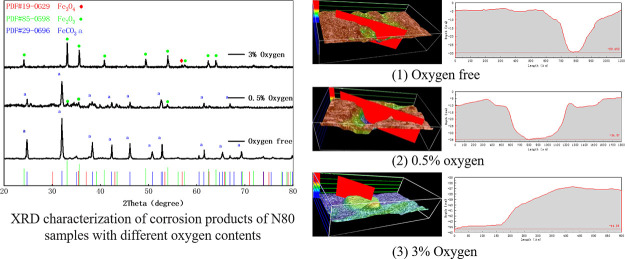

In order to investigate the corrosion behavior of N80 steel in production wellbores of oxygen-reduced air drive, the main corrosion control factors are analyzed based on gray relational analysis. Taking reservoir simulation results as indoor simulation parameters, the corrosion behavior in different production periods is studied by the dynamic weight loss method combined with metallographic microscopy, XRD, 3D morphology, and other related characterizations. The results show that oxygen content is most sensitive to the corrosion of production wellbores. The corrosion rate increases significantly under oxygen-containing conditions, and the corrosion rate at an oxygen content of 3% (0.3 MPa) is about 5 times higher than that without oxygen. At the initial stage of oil displacement, the corrosion is CO_2_-dominated localized corrosion, and the corrosion products are mainly compact FeCO_3._ With the prolongation of gas injection time, the wellbore is in a CO_2_/O_2_ balanced environment, the corrosion changes into a combined action of the two, and the corrosion products are FeCO_3_ and loose porous Fe_2_O_3_. After continuous gas injection for 3 years, the production wellbore is in a high O_2_ and low CO_2_ environment, the dense FeCO_3_ is destroyed, the corrosion pit develops horizontally, and the corrosion changes to O_2_-dominated comprehensive corrosion.

## 1 Introduction

The proven reserves in China’s low-permeability reservoirs are on the rise. Its pore throat radius is narrow. The recovery rate using conventional water injection is low. Therefore, gas flooding technology has significant advantages for the development of low permeability reservoirs.^1−6^ Compared with other gas flooding methods, oxygen-reduced air drive^7,8^ has abundant raw gas sources and low prices, and nitrogen contained in injected gas can maintain formation pressure. Oxygen undergoes a low-temperature oxidation reaction in the formation, and the released heat volatilizes light components in crude oil. It can reduce the viscosity of oil products and achieve the effect of flue gas flooding, which significantly improves the sweep efficiency and oil displacement efficiency. With the in-depth development of oxygen-reduced air drive, the corrosion of downhole string becomes more and more serious. In the process of oxygen-reduced air drive, when the gas breakthrough is fast or the oxidation reaction is incomplete,^9^ the displacement front would contain O_2_ and very little CO_2_. In addition, the water content of the produced crude oil gradually increases, which accelerates the corrosion in the high temperature and high-pressure environment. There are many studies on corrosion behavior of O_2_/CO_2_ coexisting environments. Wang^10^ studied the corrosion effect of O_2_ on X80 steel in the presence of CO_2_ through electrochemical experiments. It was found that when the oxygen partial pressure increased, the average corrosion rate increased, and the corrosion product film was destroyed, resulting in local corrosion of metal. Yuan et al.^11^ introduced the corrosion behavior of Cr steel in the multi-thermal fluid environment where O_2_/CO_2_ coexisted and found that 3Cr steel had serious corrosion. The corrosion product film was mainly composed of Fe_3_O_4_ and FeCO_3_, and there were clear cracks, which could not block the entry of corrosive media. Xueqiang et al.^12,13^ studied that the corrosion rate of N80 steel first increased sharply and then decreased slowly with the increase of temperature, and reached its peak at 90 °C. The corrosion product film of P110 steel in O_2_/CO_2_ co-existing environments has a double-layer structure, and the corrosion rate is much higher than that in single-gas environments. In view of the wellbore corrosion of oxygen-reduced air drive, Jianpeng et al.^14^ explained the oxygen corrosion of oxygen-reduced air drive of injection wellbore and found that when the oxygen-reduced value of air injection is 5%, both production cost and corrosion requirements can be taken into account. Guangzhi et al.^15^ showed that the injection well can meet the oilfield corrosion protection control index (0.076 mm/a) under the condition that the reservoir temperature is lower than 120 °C and there is no water. Compared with gas injection wells in pure gas environments, the corrosion of production wellbores is much more serious. Almeida et al.^16^ investigated the electrochemical corrosion characteristics of metals in the presence of CO_2_ and came up with an updated dissolution mechanism. In the study of oxygen reduction air drive in production wells, there are fewer studies on corrosion for high-temperature and high-pressure oil–water mixed environments. There are also fewer corrosion studies for high oxygen and low CO_2_ environments. In order to carry out oxygen reduction air drive, the main control factors of corrosion in production wells were clarified by gray correlation analysis. Weight loss experiments combined with morphological analysis were carried out to provide some data support for corrosion protection of oxygen reduction air drive production wells.

## 2 Analysis of Main Control Factors

The produced fluid of oxygen-reduced air drive has the characteristics of high salinity, high oxygen content, and carbon dioxide content, which leads to the complex wellbore situation. The gray relational grade analysis method^17−19^ can measure the correlation degree among various influencing factors. Construct the original matrix based on the data in a pilot experimental well of oxygen-reduced air drive in China. The experimental data are shown in Table 1. The matrix is treated dimensionless, and the correlation coefficient and correlation degree between corrosion rate (reference sequence) and corrosion influencing factors (comparison sequence) are calculated according to eqs 1 and 2, respectively. The calculation results are shown in Table 2. According to the principle of gray relational analysis, oxygen content is the most sensitive to corrosion. In the production process of oxygen-reduced air drive, the oxygen content at the output end should be monitored and controlled emphatically.
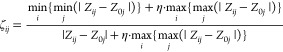
1where ζ_*ij*_ is the correlation coefficient; *Z_ij_* is the dimensionless value of the *j* index of the *i* object in the sample; and η is the resolution coefficient, taking 0.5:
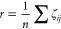
2where *r* is the correlation degree and *n* is the number of sample objects.

**Table 1:** Original Matrix of the Gray Relational Grade Model

no.		reference sequence	comparison sequence							
	standard deviation	Y	X1	X2	X3	X4	X5	X6	X7	X8
		average corrosion rate/(mm/a)	oxygen content/%	carbon dioxide content/%	K^+^ + Na^+^/mg/L	Ca^+^/mg/L	Mg^2+^/mg/L	Cl^–^/mg/L	HCO_3_^–^/mg/L	total salinity/mg/L
1	0.00108	0.2845	2.99	0.53	13290.6	10767.1	1845.2	44837.2	134.5	70874.6
2	0.00176	0.2684	2.87	0.45	15399.1	9098.1	1683.8	42320.4	143.8	68645.2
3	0.00103	0.2789	3.02	0.38	15908.3	9291.0	1788.5	42840.9	148.1	69976.8
4	0.00189	0.2705	3.12	0.45	12070.5	9595.8	1704.8	44635.3	144.9	68151.3
5	0.00229	0.2738	3.14	0.48	12804.9	10373.7	1737.6	48253.6	138.6	73308.4
6	0.00162	0.2709	2.96	0.51	14631.5	9870.2	1709.2	46182.6	153.6	72547.1
7	0.00118	0.2709	2.81	0.53	14215.6	9978.3	1708.5	44324.2	163.5	70390.1
8	0.00223	0.2607	2.79	0.59	16098.3	9956.9	1607.4	45213.3	146.9	73022.8
9	0.00177	0.2347	3.01	0.49	13719.8	10029.8	1798.2	44374.5	143.3	70065.6

**Table 2:** Correlation Degree of Various Corrosion Influencing Factors

influencing factors	correlation degree	correlation degree sorting	influencing factors	correlation degree	correlation degree sorting
oxygen content (X1)	0.7817	1	Mg^2+^ (X5)	0.6889	7
carbon dioxide content (X2)	0.6984	6	Cl^–^ (X6)	0.7281	4
K^+^ + Na^+^ (X3)	0.6187	8	HCO_3_^–^ (X7)	0.7178	5
Ca^+^ (X4)	0.7375	3	total salinity (X8)	0.7415	2

## 3 Simulation Experiment of Production Wellbore Corrosion of Oxygen-Reduced Air Drive

### 3.1 Experimental Conditions

**Figure 1:**
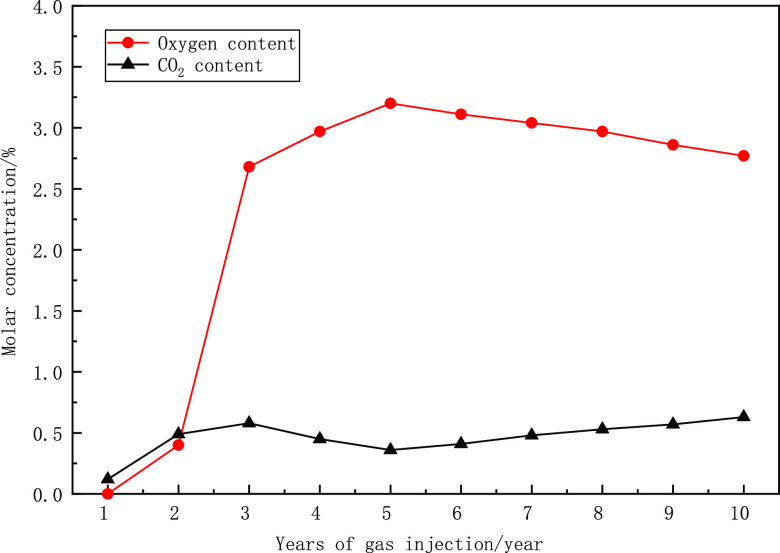
Gas composition of produced liquid with 5% oxygen-reduced air injection.

A domestic oilfield uses 5% oxygen-reduced air for oil displacement. The gas composition of simulated produced fluid in this oilfield reservoir is shown in Figure 1. The oxygen content at the output end increases significantly at first, and after continuous injection for 3 years, the oxygen content is stable at 3%. In order to make the indoor simulation experiment more close to the actual working conditions, oxygen content of 0–3% (0–0.3 MPa), temperature of 70 °C, pressure of 10 MPa, and CO_2_ content of 0.5% (partial pressure 0.05 MPa) of the 10 year simulation results were selected as the experimental working conditions. The actual oilfield recovery is used as the simulation medium. The ionic components of the simulated medium is the average value of each ionic component of the actual oilfield recovery fluid in Table 1. The dissolved content of gas in solution and the partial pressure on the surface of solution follow Henry’s law.^20,21^ The oxygen content in the production wellbore is simulated by controlling the partial pressure of the gas, the temperature and pressure environment in the production wellbore is simulated by the automatic temperature control function of the high temperature and high-pressure corrosion simulator and the closed pressure bearing property, and the phase mixing in the wellbore is realized by magnetic rotor stirring.

### 3.2 Experimental Materials and Procedures

The experiments were conducted using N80 corrosion specimens provided by Yangzhou Xingwei Machinery Company. The main chemical compositions are as follows: C 0.47%, Si 0.25%, Mn 1.65%, P 0.015%, S 0.008%, Nb 0.06%, Ti 0.03%, Mo 0.05%, Cr 0.18%, Ni 0.18%, Cu 0.18%, and Fe balance.

**Figure 2:**
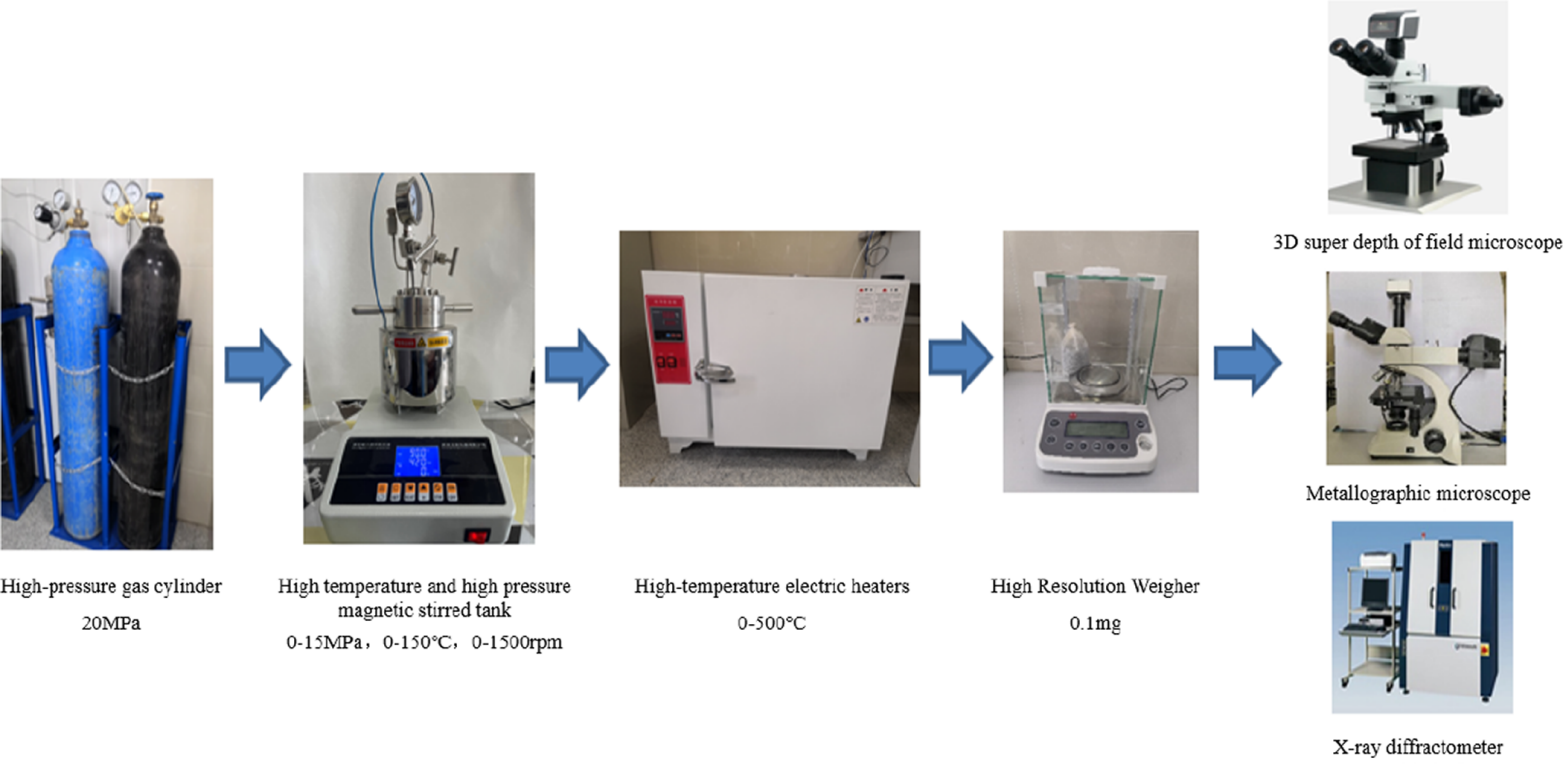
Experimental flow chart.

The procedure in the experimental preparation phase is as follows: First, use 400 # to 1200 # sandpaper to grind the test pieces step by step before the start of the experiment. Second, the test pieces were wiped clean with filter paper, then put into petroleum ether and anhydrous ethanol successively for de-oiling and dehydration. Then, put the polished test pieces into a drying oven for drying. Then, measure the length, width, height, and aperture size of the test pieces (accurate to 0.02 mm), weigh them (accurate to 0.1 mg), and record the numbers. Finally, introduce high-purity nitrogen (>99.999%) into the simulated corrosive medium to deoxidize for 12 h before the experiment. The experiment was completed in a high-temperature and high-pressure magnetic stirred tank (PFK-250ML-10 MPa/150 °C). The experimental flow is shown in Figure 2. The steps of the experiment are as follows: Step 1, pre-install the treated test piece and corrosive medium. Step 2, open the gas inlet valve and the gas outflow valve of the reaction kettle at the same time, slowly introduce high-purity nitrogen for 30 min, and remove the oxygen in the experimental pipeline and the reaction kettle. Step 3, turn on the temperature control switch. Step 4, after the temperature stabilizes to the experimental temperature, introduce oxygen (>99.995%), carbon dioxide (>99.995%), and nitrogen(>99.999%) into the kettle to the target pressure (10 MPa), the gases used in the experiments were provided by Wuhan Lanjiang Gas Company. Step 5, set the experimental time for 168 h, and open the magnetic stirring switch to start the experiment.

After the experiment, take out the test piece, use filter paper to absorb the water on the surface of the test piece, and observe the macroscopic corrosion morphology on the surface of the test piece. Then, put the test piece into pickling solution (500 mL hydrochloric acid + 3.5 g hexamethylenetetramine + 500 mL distilled water) for ultrasonic cleaning for 10 min, take it out, dehydrate, dry, and weigh. In order to avoid the experimental error caused by mechanical loss, in the cleaning process, a group of blank test pieces are taken to carry out this step synchronously, and the weightlessness of blank test pieces before and after cleaning is recorded. The average corrosion rate was calculated by eq 3, the surface corrosion morphology of the specimen was observed by an XQTD-HJXI metallographic microscope, the pitting corrosion of the specimen was measured by LY-WN-YH 3D super depth of field microscope, and the corrosion products were tested by XRD by Rigaku Ultima IV X-ray diffractometer:
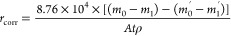
3where *r*_corr_ is the corrosion rate (mm/a); *m*_0_ is the pre-test mass (g) of corrosion test piece; *m*_1_ is the quality of corrosion test piece after cleaning (g); *m*_0_ is the quality of blank test piece before cleaning (g); *m*_1_ is the quality (g) of the blank test piece after cleaning; *A* is the total surface area of the sample (cm^2^); ρ is the density of sample material (g/cm^3^); and *t* is test time (h).

## 4 Results and Discussion

### 4.1 Corrosion Rate

**Figure 3:**
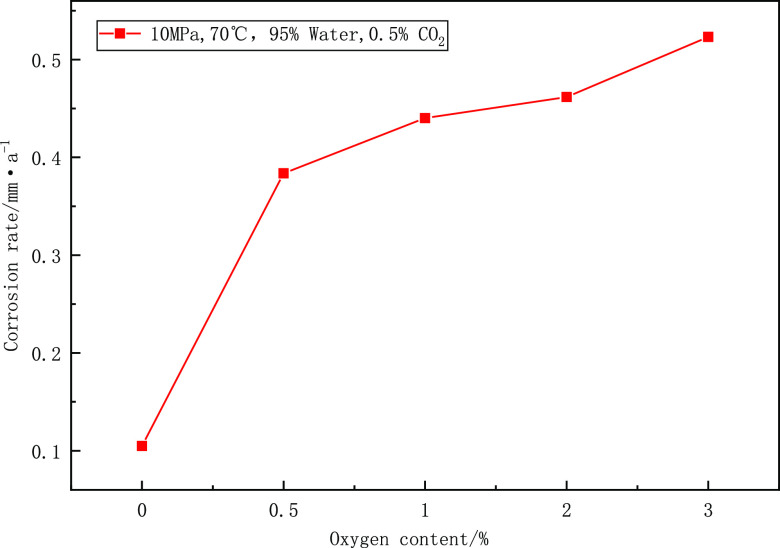
. Corrosion rate of N80 specimen under different oxygen contents.

Test results of the corrosion rate of N80 specimens with different oxygen content are shown in Figure 3 under the working conditions of 70 °C, 10 MPa, 95% water, and 0.5% CO_2_. It can be seen from the figure that the corrosion rate of N80 steel increases significantly from no oxygen to 0.5% oxygen, with a corrosion growth rate of 266%, from 2 to 3% oxygen, with a corrosion growth rate of 13%, and the corrosion rate growth rate slows down obviously. The uniform corrosion rate at 3% oxygen is 0.5232 mm/a, which is about 5 times higher than that without oxygen content. According to NACE SP 0775-2013 corrosion evaluation index, the corrosion is extremely serious (>0.254 mm/a) under oxygen-containing conditions, and moderate (0.025–0.12 mm/a) without oxygen. Reservoir simulation results show that the production wellbores are in the environment of high oxygen content and low carbon dioxide for a long time in the later period of gas injection, so the production wellbores of oxygen-reduced air drive face high corrosion risk.

### 4.2 Macroscopic Morphology

**Figure 4:**
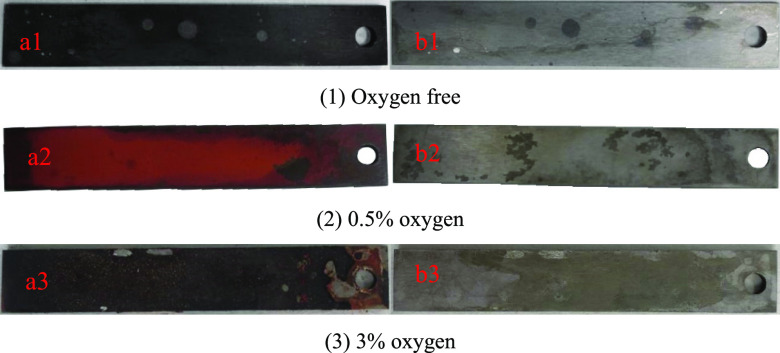
Macroscopic corrosion morphology of specimens before and after removing corrosion products under different oxygen contents.

From the surface morphology of the N80 specimen before and after cleaning, it can be seen from Figure 4 that with the increase of oxygen content, the corrosion product layer on the surface of the specimen gradually increases and thickens, which is consistent with the weight loss result. Without oxygen, the corrosion products on the surface of the specimen are black, uniformly attached to the surface of the specimen, and closely combined with the substrate. After pickling, the surface of the specimen is flat and still shows a certain metallic luster. There are many elliptical scars on the surface of the specimen before and after pickling. From the point of view of microdroplets,^22−24^ the high-speed motion of the magnetic rotor simulates the phase mixing in the lifting process of production wells, and the emulsion is oil-in-water type, and the scar is caused by the adhesion of oil droplets. Under 0.5% oxygen condition, the corrosion products on the surface of the specimen are reddish brown and black, with fine structure and no obvious shedding. After pickling, the metallic luster of the surface of the specimen is weakened, and the red-brown corrosion products cover the middle and edge, and obvious corrosion areas appear. Under the condition of 3% oxygen content, the corrosion products have two layers, the inner layer is reddish brown and the outer layer is black, and the structure is loose and easy to fall off. Before pickling, part of the matrix was exposed, and the corrosion was obvious after pickling.

**Figure 5:**
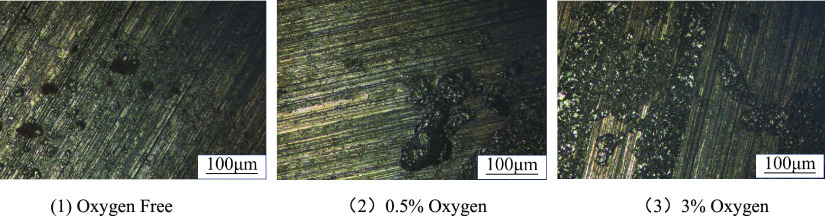
Surface corrosion morphology of N80 samples after cleaning under different oxygen contents.

The corrosion morphology of the N80 specimen after cleaning is observed by a metallographic microscope as shown in Figure 5. When oxygen is not contained, the surface corrosion of the specimen is slight, the mechanical scratches are obviously exposed, only a few shallow corrosion pits exist and the corrosion areas are scattered. When the oxygen content increases to 3%, the corrosion area on the sample surface expands, and the corrosion area changes from dispersion to aggregation.

### 4.3 Microscopic Morphology

**Figure 6:**
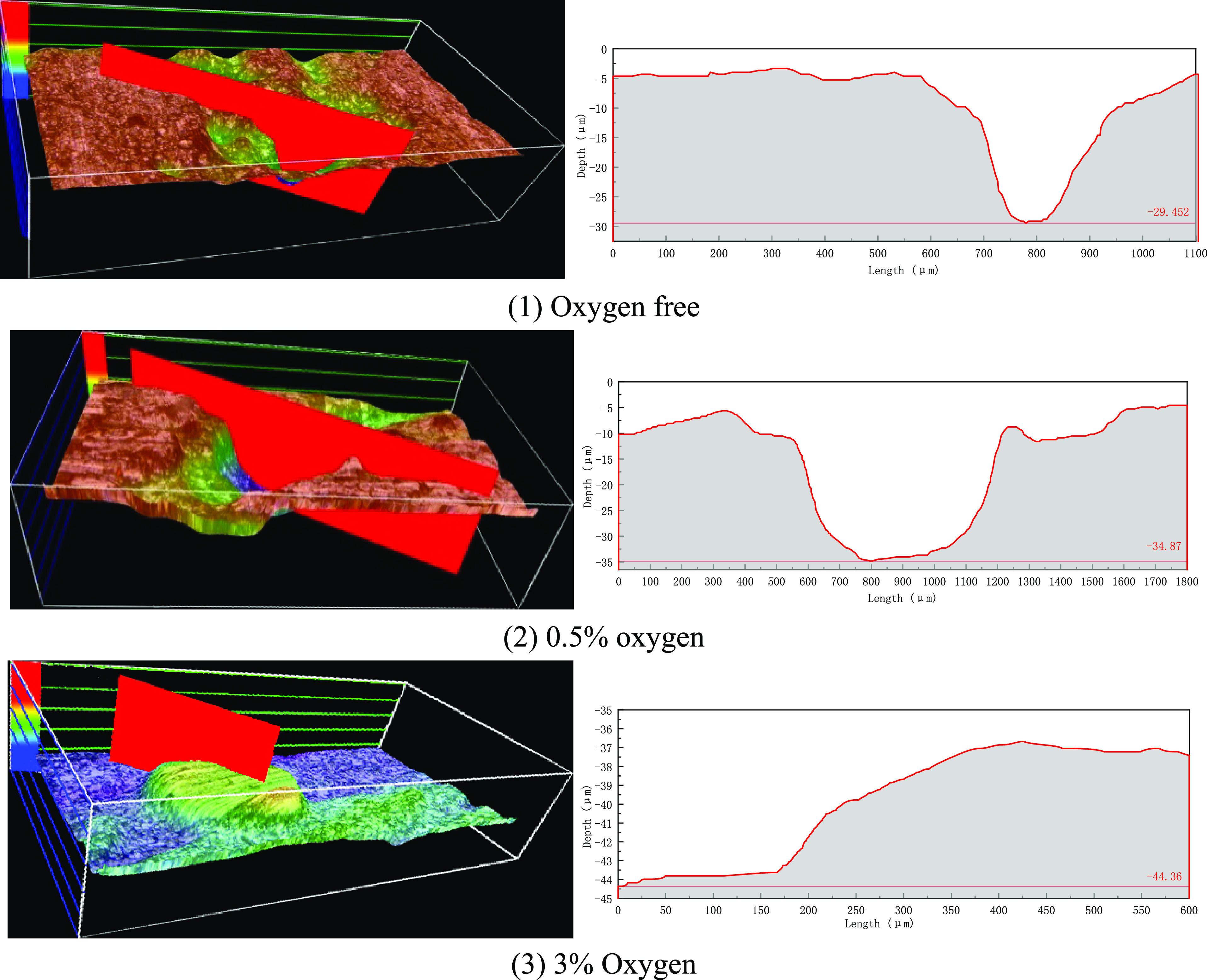
3D morphology and size of N80 samples under different oxygen contents.

3D models of N80 specimens with different oxygen contents were observed by using an ultra-depth of field 3D microscope, as shown in Figure 6. In the absence of oxygen, the surface of the specimen is flat, and there is a little local corrosion. The morphology of corrosion pits is mainly narrow and deep, and the maximum depth of corrosion pits is 29.45 μm. The ratio of maximum corrosion depth to average corrosion depth (local corrosion coefficient) is used to reflect the degree of local corrosion.^25^ In the absence of oxygen, the local corrosion coefficient is 13.28, which belongs to serious local corrosion; Under the condition of 0.5% oxygen content, the corrosion pits develop transversely and change into elliptical ones. The maximum depth of corrosion pits is 34.87 μm, which is smaller than that without oxygen, but the width of corrosion pits increases by nearly 2.5 times. Under the condition of 3% oxygen content, the overall corrosion of the specimen is serious. The maximum corrosion depth is 44.36 μm, the local corrosion coefficient is 2.54, and the corrosion changes from local corrosion to comprehensive corrosion.

**Figure 7:**
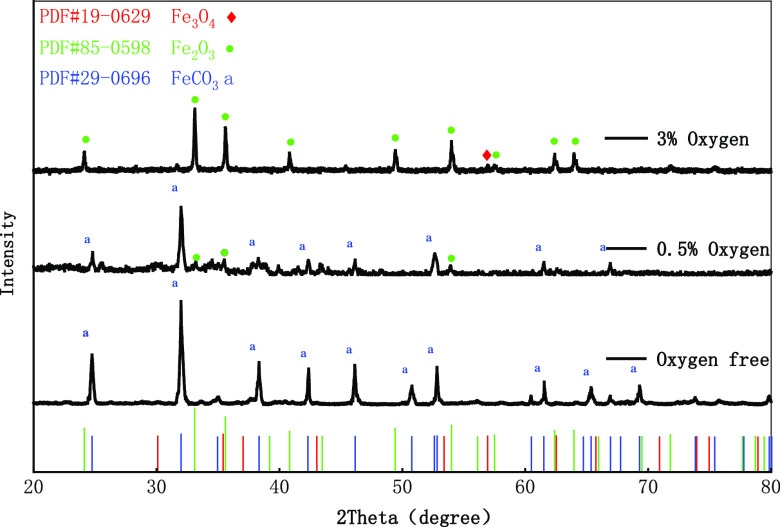
XRD characterization of corrosion products.

After grinding and sieving the corrosion products in typical corrosion parts of the specimens, XRD analysis was carried out, and the diffraction peaks of corrosion products under different oxygen contents were obtained by Jade software analysis. By comparing the phase composition of corrosion products obtained by standard pdf cards, Figure 7 shows XRD test results under 0, 0.5, and 3% oxygen. Corrosion products are different under different oxygen content, mainly including Fe_2_O_3_, Fe_3_O_4_, and FeCO_3_. Under the condition of no oxygen, only the diffraction peak of FeCO_3_ was detected. The temperature and pressure environment of the production wellbore is simulated experimentally, and FeCO_3_ is in the initial stage of deposition at 70 °C.^26^*c*(Fe^2+^) × *c*(CO_3_^2–^) in the solution is just larger than Ksp(FeCO_3_), and the precipitated FeCO_3_ covers the substrate surface. However, due to the problem of insufficient temperature and CO_2_ content,^27^ the structure of precipitated FeCO_3_ is not stable enough, and local pitting corrosion occurs in uncovered areas, which is consistent with the depth of field results. According to the intersection of O_2_ content and CO_2_ content in Figure 1, it can be seen that O_2_ and CO_2_ reach equilibrium at 0.5% oxygen content. In addition, the corrosion products are FeCO_3_ and a small amount of Fe_2_O_3_. When the oxygen content increases to 3%, the corrosion products are only two oxides of iron. Combined with macroscopic morphology, the corrosion products of the inner layer are Fe_2_O_3_ and the outer layer are Fe_3_O_4_.

### 4.4 Corrosion Mechanism

Combined with the color change of the specimen surface under macroscopic morphology and XRD analysis results, at the initial stage of gas injection, the oxidation reaction is complete, and there is almost no O_2_ in the production wellbore for the production wellbores flooded with 5% oxygen-reduced air. CO_2_ corrosion plays a leading role. CO_2_ first undergoes hydrolysis and ionization and reacts with Fe and Fe(OH)_2_ to form FeCO_3_. As shown in eqs 4 and 5, FeCO_3_ has a compact structure and covers the metal surface, and the corrosion rate is small at this time. With the prolongation of gas injection time, the content of O_2_ in the wellbore increases, and the corrosion rate increases significantly under the joint action of O_2_ and CO_2_. The strong oxidization of O_2_ promotes the corrosion of N80, preferentially oxidizing Fe^2+^ to Fe(OH)_3_, and Fe(OH)_3_ produces FeO(OH) after the dehydration reaction, and further dehydrates to produce Fe_2_O_3_. As shown in eq 6, the iron oxide formed in this stage has loose and porous structure and poor protection to the matrix:
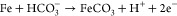
4
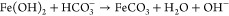
5
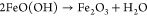
6

Reservoir simulation results show that after continuous gas injection for 3 years, the oxygen content at the output end is stable at 3%. The content of O_2_ reaches 6 times of CO_2_, and O_2_ corrosion plays a leading role. The protective film of FeCO_3_ formed in the early stage is destroyed, and FeCO_3_ reacts with oxygen to form unprotected Fe_2_O_3_. As shown in eq 7, corrosion products are stacked on the metal surface, and the covered area forms an occluded microenvironment. The original corrosion pits develop horizontally, and at the same time, new corrosion pits will be generated, resulting in intensified corrosion. In addition, with the increase of oxygen content, the reaction occurs further, and FeO(OH) reacts with Fe^2+^ to form Fe_3_O_4_, as shown in eq 8, which also cannot inhibit the occurrence of corrosion. At the initial stage of gas injection in oxygen-reduced air drive, CO_2_ corrosion should be emphasized, and anti-corrosion measures such as adding an anti-CO_2_ corrosion inhibitor should be selected. With the extension of gas injection time, when the produced fluid gas is detected to be stable at high O_2_ and low CO_2_, an anti-oxygen corrosion inhibitor should be added instead to achieve a good anti-corrosion effect:
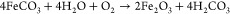
7
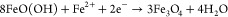
8

## 5 Conclusions

(1) The produced fluid of oxygen-reduced air drive has the characteristics of high salinity, high oxygen content, and carbon dioxide content, which leads to complex conditions of production wellbores. According to gray correlation theory, it is clear that oxygen content has the most prominent influence on corrosion of production wellbores.

(2) Based on the dynamic corrosion simulation experiment, the corrosion rates of N80 specimens in different production periods were investigated. The results showed that the corrosion rates increased nearly 5 times when the oxygen content increased from 0 to 3%, and the corrosion rates increased slowly with the continuous increase of oxygen content. Macroscopic morphology shows that with the increase of oxygen content, corrosion products increase and thicken, and corrosion areas change from dispersion to aggregation.

(3) XRD test and 3D depth of field topography were used to characterize the N80 specimen. With the prolongation of oil displacement time, the corrosion changed from CO_2_-dominated to CO_2_/O_2_-dominated and then to O_2_-dominated. Corrosion pits mainly develop horizontally, from local corrosion to comprehensive corrosion. The corrosion products changed from FeCO_3_ to Fe_2_O_3_ and Fe_3_O_4_, which did not protect the substrate but made the area covered by the products form an occluded microenvironment, which led to the aggravation of corrosion. Therefore, different corrosion inhibition measures should be considered for different periods of gas injection in oxygen-reduced air drive.
